# Physician Communication, Repeated Healthcare Encounters, and Diagnostic Delay in Endometriosis: An Exploratory Cross-Sectional Analysis

**DOI:** 10.3390/healthcare14193237

**Published:** 2026-10-01

**Authors:** Cristina-Elena Mandici, Daniela Roxana Matasariu, Irina Draga Căruntu, Bogdan Doroftei, Daniela-Cristina Dimitriu, Liliana-Claudia Strat, Lucian Vasile Boiculese, Alexandra Ursache, Iuliana-Elena Bujor, Ioana-Adriana Popescu, Mihaela Grigore

**Affiliations:** 1 Grigore T. Popa University of Medicine and Pharmacy Iasi, 700115 Iasi, Romania; cristina-elena.mandici@umfiasi.ro (C.-E.M.); irina.caruntu@umfiasi.ro (I.D.C.); bogdan.doroftei@umfiasi.ro (B.D.); daniela.dimitriu@umfiasi.ro (D.-C.D.); liliana.strat@umfiasi.ro (L.-C.S.); vasile.boiculese@umfiasi.ro (L.V.B.); alexandra.ursache@umfiasi.ro (A.U.); iuliana_elena.bujor@umfiasi.ro (I.-E.B.); ioana-adriana.popescu@umfiasi.ro (I.-A.P.); mihaela.grigore@umfiasi.ro (M.G.); 2 Cuza Voda Clinical Hospital Obstetrics and Gynecology, 700038 Iasi, Romania; 3 Department of Morpho-Functional Sciences I—Histology, “Grigore T. Popa” University of Medicine and Pharmacy, University Street 16, 700115 Iasi, Romania; 4 Department of Biochemistry, “Grigore T. Popa” University of Medicine and Pharmacy, 16 Universitatii Street, 700115 Iasi, Romania; 5 Biostatistics, Department of Preventive Medicine and Interdisciplinarity, “Grigore T. Popa” University of Medicine and Pharmacy, 700115 Iasi, Romania; 6 Department of Dermatology, “Grigore T. Popa” University of Medicine and Pharmacy, 700115 Iasi, Romania

**Keywords:** endometriosis, diagnostic delay, patient–physician communication, care fragmentation, mediation analysis, adolescent onset, symptom minimization, women’s health

## Abstract

**Highlights:**

**What are the main findings?**

- In 150 women with self-reported, surgically confirmed endometriosis, diagnosis was established a mean of 9.1 years after symptom onset; symptom severity was not associated with this delay, whereas an earlier (adolescent) symptom onset identified those at highest risk.
- Poor patient–physician communication was associated with delay indirectly, through a higher number of pre-diagnostic consultations (repeated healthcare encounters), rather than directly; because the design is cross-sectional, this indirect association is exploratory.

**What are the implications of the main findings?**

- Adolescents with severe dysmenorrhea warrant a lower threshold for suspecting endometriosis, since they are at greatest risk of a prolonged diagnostic delay.
- Reducing repetitive, unproductive consultations, for example through structured pre-consultation symptom tools, may be a testable strategy for shortening diagnostic delay.

**Abstract:**

Background: Endometriosis is often diagnosed years after symptom onset, while communication-related factors remain poorly defined in Central and Eastern Europe. Methods: In this cross-sectional study, 150 Romanian women reporting surgically confirmed endometriosis completed an online questionnaire on diagnostic history and patient–physician communication, assessed with a seven-item Likert scale (Cronbach’s α = 0.836). Results: Mean diagnostic delay was 9.1 years (median 8; IQR 4–13), with 5.2 physicians and 10.2 consultations before diagnosis. Delay was unrelated to symptom severity (r = 0.07, *p* = 0.43) but inversely related to age at symptom onset (r = −0.68). Adolescent-onset symptoms were followed by a 14.9-year delay versus 5.8 years for adult-onset symptoms. In multivariable analysis (adjusted R^2^ = 0.49), earlier onset and more consultations remained independently associated with delay. Communication had an indirect association through consultation number (effect −0.68; 95% bootstrap CI −1.26 to −0.22), accounting for 38% of the total association; direction cannot be inferred from cross-sectional data. Symptom minimization (76%) was linked to more physicians and longer delays (10.0 vs. 6.0 years; d = 0.60). Conclusions: Earlier onset and repeated healthcare encounters were related to delay than symptom severity. The self-selected, urban, highly educated sample limits generalizability and causal interpretation.

## 1. Introduction

Endometriosis is a chronic, estrogen-dependent inflammatory condition defined by the presence of endometrium-like glandular and stromal tissue outside the uterine cavity, and it affects approximately 10% of women and girls of reproductive age worldwide [1]. The ectopic lesions most frequently involve the ovaries, the pelvic peritoneum, the uterosacral ligaments, the rectovaginal septum, and the bowel, and they are conventionally grouped into three phenotypes: superficial peritoneal endometriosis, ovarian endometrioma, and deep infiltrating endometriosis [2,3]. Although the disorder is histologically benign, its clinical evolution is often progressive, and the burden it imposes on physical, reproductive, and psychosocial health is considerable [1,2].

Its clinical presentation is markedly heterogeneous: alongside the cardinal features of pelvic pain and subfertility, women commonly report dysmenorrhea, deep dyspareunia, dyschezia, dysuria, cyclical gastrointestinal or urinary complaints, chronic fatigue, and a high prevalence of anxiety and depressive symptoms [1,4]. This diversity of manifestations, together with their overlap with other gynecological and gastrointestinal disorders, contributes to the difficulty of recognizing the disease at an early stage [2,5].

The pain of endometriosis is not explained by lesion burden alone. Persistent nociceptive input can drive peripheral and central sensitization, giving rise to neuropathic and nociplastic pain that may persist independently of hormonal cyclicity and of the anatomical extent of disease [6]. This dissociation between symptom severity and observable pathology helps to explain why clinical assessment is challenging and why patient-reported experience is central to timely recognition [6].

A well-documented feature of endometriosis is the prolonged interval between symptom onset and diagnosis, which is commonly reported to range from four to eleven years [4,7]. Symptoms frequently begin during adolescence, and many women consult several clinicians across different specialties before the condition is identified [7,8]. This delay carries clinical, psychological, and economic consequences: it is associated with disease progression, repeated and sometimes unnecessary investigations or procedures, prolonged pain, impaired fertility, and substantial psychosocial and economic costs, including reduced educational attainment and work productivity [3,4].

For many years, laparoscopy with histological confirmation was regarded as the diagnostic reference standard. Current guidelines, however, support a clinical diagnosis based on symptom history, pelvic examination, and imaging, particularly transvaginal ultrasound and magnetic resonance imaging when performed by experienced operators, reserving surgery for selected cases [2,3]. Structured sonographic protocols such as the International Deep Endometriosis Analysis (IDEA) consensus have improved the standardization of imaging assessment [9]. Efforts to identify accurate non-invasive biomarkers continue, but no circulating or tissue marker has yet demonstrated sufficient diagnostic accuracy for routine clinical use [10].

Even with these advances, early diagnosis remains elusive because presenting symptoms are non-specific and overlap with adenomyosis, irritable bowel syndrome, and primary dysmenorrhea, so that endometriosis is often not considered at first contact [2,7].

In this context, the quality of patient–physician communication has emerged as a potentially modifiable element of the diagnostic pathway. Effective, patient-centered communication, characterized by active listening, empathy, and clear information exchange, supports accurate symptom reporting and earlier suspicion of the disease [11]. Conversely, a substantial volume of qualitative and mixed-methods research shows that women with endometriosis frequently feel dismissed or disbelieved, describe having their pain normalized or attributed to psychological causes, and struggle to translate the perceived pain into language that clinicians can act on [4,11,12]. How such experiences translate into a longer time to diagnosis, however, remains unclear. One plausible route is that, when their concerns are not addressed, women seek repeated opinions, so that the diagnostic pathway becomes longer and more fragmented [4,8]; whether the number of consultations accounts for the association between communication and delay has not, to our knowledge, been formally tested.

**Figure 1:**
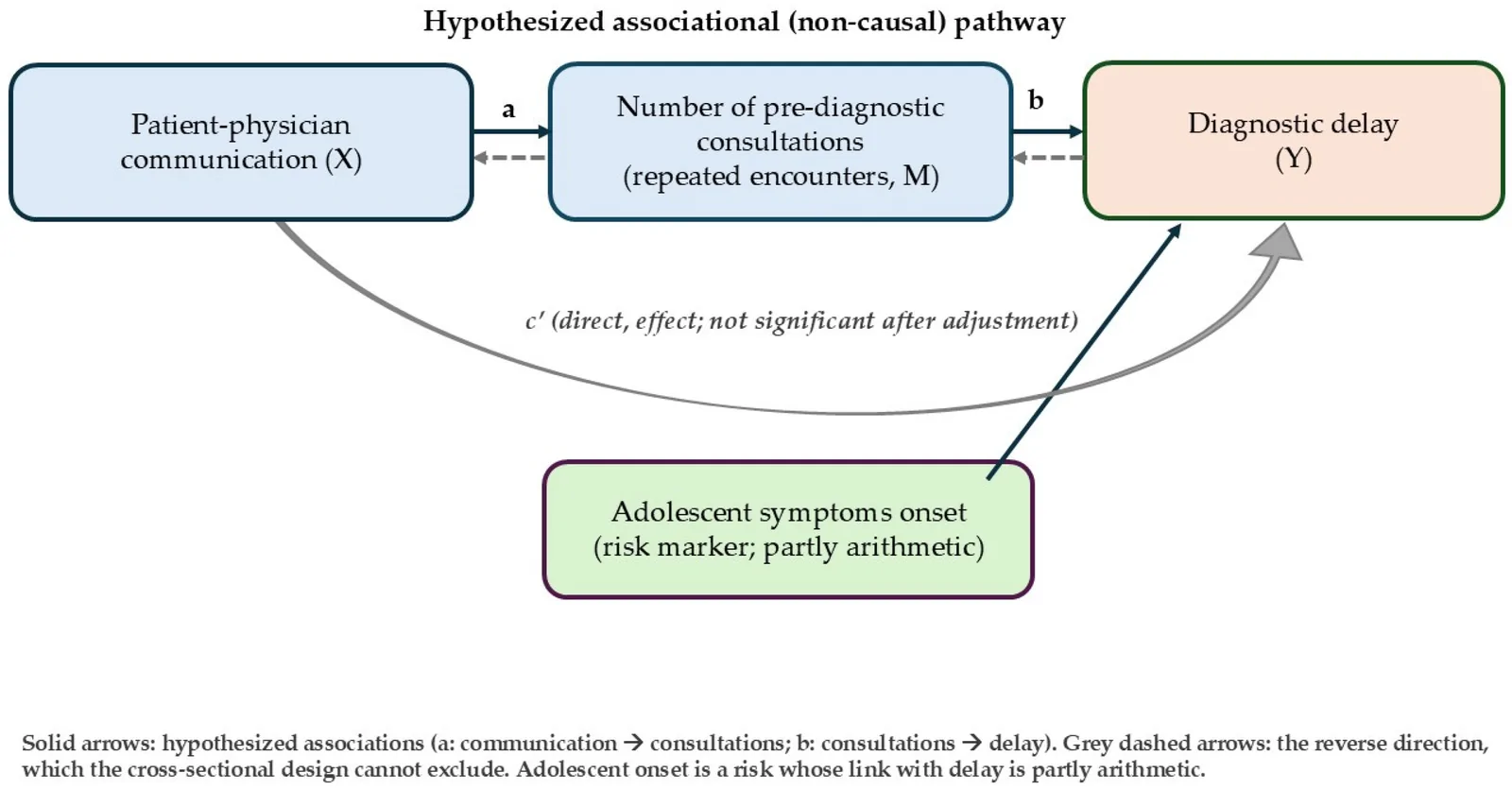
Hypothesized associational (non-causal) pathway linking patient–physician communication, the number of pre-diagnostic consultations (repeated healthcare encounters), and diagnostic delay. Arrows denote hypothesized statistical associations, not established causal effects; because the data are cross-sectional, the direction of the a and b paths cannot be determined. Adolescent symptom onset is shown as a risk marker whose association with delay is partly arithmetic.

Poor communication is frequently cited as a cause of the long delay in diagnosing endometriosis, yet whether and how it lengthens the diagnostic pathway remains unclear. Evidence from Central and Eastern Europe, including Romania, is also scarce. Because symptom severity did not predict how long women waited in this cohort, we examined which women were most at risk and the route through which communication might act. Specifically, the present study had three aims: to characterize the diagnostic pathway of Romanian women with endometriosis; to identify factors independently associated with diagnostic delay, including age at symptom onset, and to examine whether the number of consultations before diagnosis is statistically consistent with an indirect, associational pathway linking patient–physician communication and delay. This work forms part of a doctoral research project conducted at the “Grigore T. Popa” University of Medicine and Pharmacy, Iași, Romania. We addressed three research questions: (i) how long is the diagnostic pathway of Romanian women with endometriosis, and which factors are associated with its length; (ii) is patient–physician communication associated with diagnostic delay; and (iii) is any such association statistically consistent with an indirect pathway operating through the number of pre-diagnostic consultations? We hypothesized that poorer communication would be associated with a longer delay and that this association would be at least partly indirect, operating through a higher number of consultations; given the cross-sectional design, these hypotheses were framed as associational rather than causal (Figure 1).

## 2. Materials and Methods

### 2.1. Study Design and Reporting

This was an observational, cross-sectional study based on an anonymous online survey of women with endometriosis in Romania, conducted as part of a doctoral research project at the “Grigore T. Popa” University of Medicine and Pharmacy, Iași. The study is reported in accordance with the STROBE (Strengthening the Reporting of Observational Studies in Epidemiology) statement for cross-sectional studies (Supplementary File S1). Data were collected online over a six-month period following ethical approval (24 November 2021), that is, between late 2021 and mid-2022; because the data therefore predate the present submission, the estimated delay should be interpreted with reference to that period, during which diagnostic practice has continued to evolve.

### 2.2. Participants and Recruitment

Participants were recruited over a 6-month period through Romanian online communities for women affected by endometriosis, including the Facebook groups “ENDOMETRIOZA—ROMANIA”, “Endometrioza #ÎMPREUNĂ”, “Endometrioza și Adenomioza Romania”, “Endometrioza Adenomioza SOP—Fertilitate”, “Endometriosis Support Group”, and “Endometrioza de la A la Z”, and through the patient organization Asociația Eu și Endometrioza. Participation was voluntary and anonymous, and women completed the survey after accessing the shared questionnaire link. Eligibility required an age of 18 years or older and a self-reported surgically confirmed diagnosis of endometriosis, of any stage; women younger than 18 years or without a surgically confirmed diagnosis were excluded. Because the survey was anonymous and no direct contact with participants was established, individual medical records and histopathology reports could not be verified.

**Figure 2:**
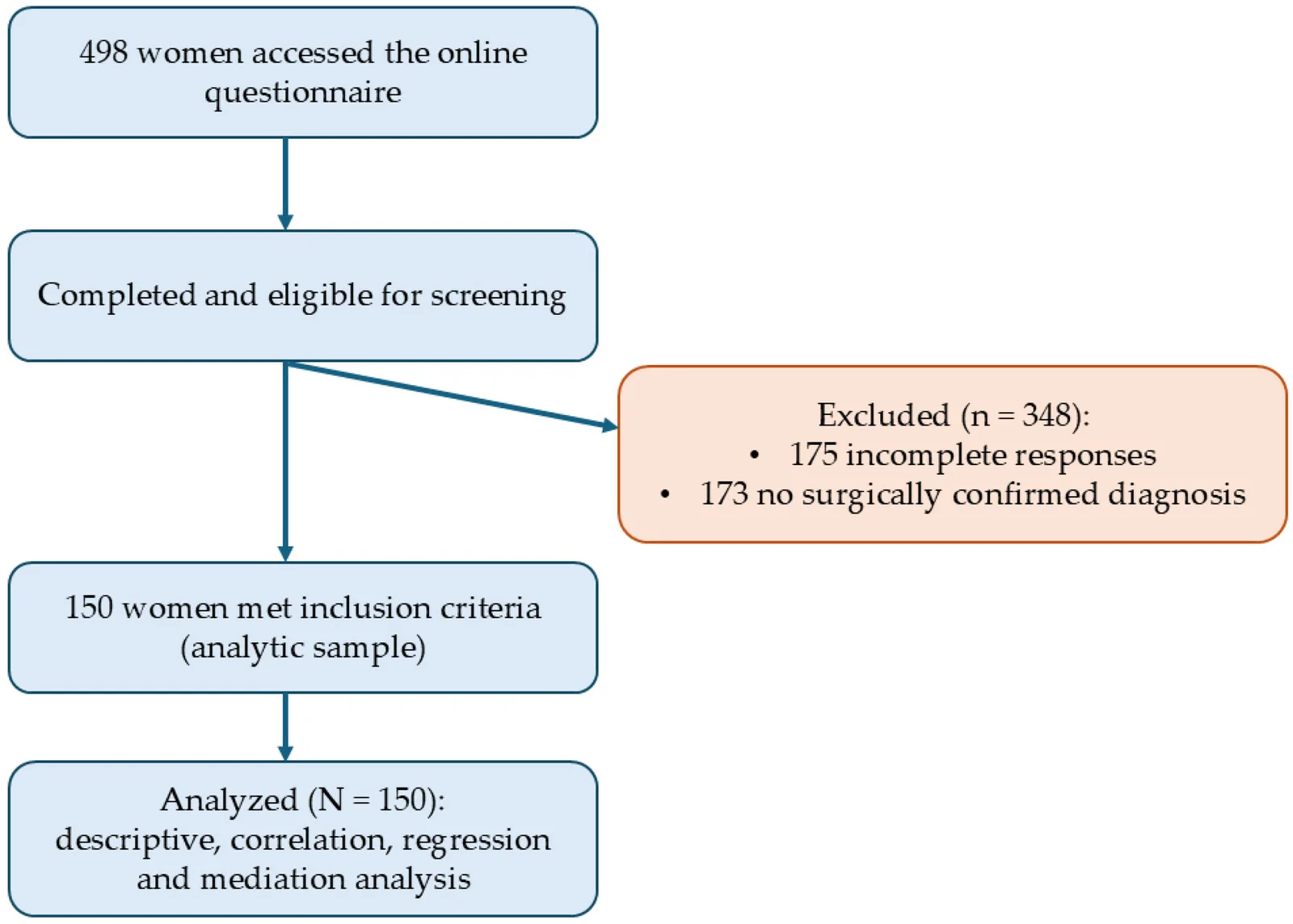
Flow of participants through the study.

Of 498 women who accessed the questionnaire, 175 did not complete it and 173 did not report a surgically confirmed diagnosis of endometriosis; the remaining 150 women met the eligibility criteria and constituted the analytic sample (175 + 173 + 150 = 498). The flow of participants is summarized in Figure 2. Because the survey was anonymous, no information was captured on women who opened but did not complete the questionnaire, so respondents and non-respondents could not be compared.

### 2.3. Questionnaire and Data Collection

Data were collected using a Google Forms questionnaire administered online in Romanian, developed by the research team specifically for this study and informed by a review of the literature on diagnostic delay [7,8] and patient–physician communication [11,12] in endometriosis; the English version in Supplementary File S2 is a translation provided for review. The final version was reviewed and approved by all investigators. The questionnaire comprised 30 closed-ended items organized in four sections: (1) demographic data (age, area of residence, and educational level); (2) medical history (symptoms, age at symptom onset, and age at diagnosis); (3) interaction with healthcare professionals (number and specialty of physicians consulted before diagnosis, the specialty establishing the diagnosis, and perceptions of communication and physician attitude); and (4) patient perception (trust in the physician, understanding of medical explanations, and perceived empathy). Communication experiences before diagnosis were rated on five-point Likert items (items 8–14); these items were presented under the heading “Experience with physicians before diagnosis” and referred to participants’ overall pre-diagnostic experience with physicians as a whole rather than to a single consultation or to the diagnosing physician, consistent with the total number of consultations serving as the mediator. The two negatively worded items (11 and 12) were reverse-coded before computing the composite score. Informed consent was obtained electronically in the first section of the form, before any study item was presented, and responses were exported to a spreadsheet for analysis. All closed-ended items were required, so the 150 analyzed questionnaires had complete data on closed items; the age items were checked for internal consistency, and the resulting diagnostic delay ranged from 0 to 31 years with no negative values, confirming that no record showed symptom onset after diagnosis. The full questionnaire is provided as an English translation in Supplementary File S2.

The items were generated by the research team from the literature on patient–physician communication in endometriosis [11,12] and reviewed and approved by the multidisciplinary investigator team, comprising practicing gynecologists and a biostatistician, as an internal content- and face-validity step; they were not additionally reviewed by an external expert panel or by patient representatives, and no separate pilot study was conducted. The questionnaire did not undergo formal external psychometric validation; good internal consistency does not establish construct validity, and the scale may partly capture a general negative evaluation of a prolonged diagnostic journey rather than communication alone; its internal consistency was therefore assessed in the present sample (see Section 2.5), and the findings should be regarded as exploratory and hypothesis-generating.

### 2.4. Variables and Derived Measures

Diagnostic delay, the primary outcome, was defined as the interval in years between the age at symptom onset and the age at diagnosis. Because this outcome is computed arithmetically from age at symptom onset, age at onset is not treated as an independent determinant of delay but as a marker identifying women at higher risk, and dedicated sensitivity analyses (Section 2.5) address this coupling. For sensitivity analyses, age at symptom onset was additionally categorized as adolescent onset (<18 years) or adult onset (≥18 years). The diagnostic pathway was characterized by the number of physicians consulted and the number of consultations attended before diagnosis, and by the specialty that established the diagnosis. Symptom severity was measured on a single 0–10 numerical rating item capturing the overall retrospective impact of symptoms on daily life during the pre-diagnostic period (questionnaire item 7); this single item does not distinguish current, average, or maximum severity, nor the clinical recognizability of symptoms. A composite communication score was computed from the seven communication items (items 8–14); the two negatively worded items were reverse-coded so that higher scores indicated a more positive communication experience, and a four-item positive subscale was also derived. For descriptive purposes, diagnostic delay was additionally dichotomized at a threshold of five years.

### 2.5. Statistical Analysis

Continuous variables were summarized as means with standard deviations and as medians with interquartile ranges; distributional shape was assessed with Skewness, Kurtosis, and the Shapiro–Wilk test. Diagnostic delay and the consultation and physician counts were right-skewed and are therefore summarized primarily with medians and interquartile ranges, whereas the composite communication score was approximately symmetric. Categorical variables were summarized as counts and percentages. The Pearson correlations and the linear model were retained because the regression residuals met the relevant assumptions rather than because each variable was marginally normal. The internal consistency of the communication scale was assessed with Cronbach’s alpha for both the seven-item scale and the four-item positive subscale. Associations between continuous variables were examined using Pearson correlation coefficients, with Spearman rank correlations computed as a sensitivity check for the skewed variables (results materially unchanged); associations between categorical variables were examined using the chi-square test. Group differences were tested with Welch’s independent-samples *t*-tests, with effect sizes expressed as Cohen’s d; differences across educational levels were assessed by one-way analysis of variance followed by Tukey HSD and Bonferroni-corrected Welch post hoc comparisons. The dimensionality of the seven-item communication scale was examined by exploratory factor analysis, with the Kaiser–Meyer–Olkin (KMO) measure and Bartlett’s test of sphericity used to assess factorability and Horn’s parallel analysis used to determine the number of factors. To limit false positives from multiple testing, *p*-values from the primary correlation and group-comparison analyses were adjusted using the Benjamini–Hochberg false discovery rate (FDR) [13].

Factors independently associated with diagnostic delay were identified using multivariable linear regression, with age at symptom onset, number of consultations, communication score, symptom severity, and place of residence entered as covariates. These covariates were pre-specified on clinical and conceptual grounds, and no automated variable-selection procedure (for example, stepwise selection) was used. The questionnaire captured age, educational level, area of residence, main symptom category, symptom impact, age at symptom onset, age at diagnosis, and the number and broad specialty of physicians consulted, as well as the number of consultations; it did not capture endometriosis phenotype or disease extent, infertility, endometrioma or deep endometriosis, family history, prior hormonal treatment, socioeconomic status beyond education, geographical access to specialist care, or healthcare-system waiting times, so residual confounding by these factors cannot be excluded. Model adequacy was assessed through the adjusted R^2^, variance inflation factors for multicollinearity, and inspection of residuals for normality (Shapiro–Wilk test) and homoscedasticity (Breusch-Pagan test).

To examine the mechanism linking communication to delay, a mediation analysis was performed following the Baron and Kenny framework, with the number of consultations before diagnosis as the mediator; the significance of the indirect effect was evaluated using nonparametric bootstrapping (5000 resamples) with bias-corrected 95% confidence intervals [14], complemented by the Sobel test. Because the data are cross-sectional, the temporal ordering of mediator and outcome cannot be established; we therefore also examined the mediator-outcome association after adjustment and interpret the mediation model as associational rather than causal. To verify that the findings were not driven by the arithmetic relationship between age at onset and delay, sensitivity analyses were conducted by re-estimating the multivariable model without age at onset, by computing partial correlations controlling for age at onset, and by comparing adolescent-onset and adult-onset groups. Free-text responses were not subjected to formal qualitative (thematic) analysis; representative quotations are reported illustratively to contextualize the quantitative findings. These open-ended items were optional, and the percentages reported for what participants felt were most lacking refer to the proportion of participants who provided a codable free-text answer to the corresponding item. The specificity of the mediation was probed with a parallel-mediator model contrasting the number of consultations with the number of physicians, and with an alternative exposure (symptom minimization); its robustness was examined by winsorizing the consultation counts at the 95th percentile and by excluding extreme values. All analyses were performed in Python (version 3.10; NumPy, SciPy, statsmodels, and scikit-learn) and cross-checked against the original spreadsheet computations. No a priori sample-size calculation was performed; the analytic sample comprised all 150 eligible respondents (a convenience sample). With this sample size, the 95% confidence intervals for the main correlations had a half-width of about 0.15, and the bias-corrected bootstrap confidence interval for the indirect effect excluded zero. Because no *a priori* sample-size calculation was performed, we do not infer sample adequacy from statistical significance; estimates from this convenience sample, particularly for the mediation and subgroup analyses, should be regarded as exploratory and potentially unstable. Ninety-five percent confidence intervals are reported for the principal correlations (Fisher z-transformation), the main subgroup difference in diagnostic delay, the regression coefficients (Wald intervals from the model standard errors), and the indirect effect (bias-corrected bootstrap). A two-sided *p*-value < 0.05 was considered statistically significant.

### 2.6. Ethical Considerations

The study was conducted in accordance with the Declaration of Helsinki and approved by the Ethics Committee of the “Grigore T. Popa” University of Medicine and Pharmacy, Iași (Approval No. 127/24.11.2021). Participation was anonymous and voluntary, and electronic informed consent was obtained from all participants before completion of the questionnaire.

## 3. Results

The 150 participants had a median age of 35 years (range 20–57). Most lived in urban areas (82.7%) and had higher or postgraduate education (84.7%). Symptoms began at a mean age of 21.7 years, and diagnosis was established at a mean age of 30.8 years. Sociodemographic characteristics and the main features of the diagnostic pathway are summarized in Table 1.

The mean interval between symptom onset and diagnosis was 9.1 years (SD 6.9; median 8; IQR 4–13; range 0–31), and 65.3% of women waited more than five years for a diagnosis. Before diagnosis, participants had consulted a mean of 5.2 physicians (median 4; range 1–30) and attended a mean of 10.2 consultations (median 10; range 1–50). A gynecologist established the diagnosis in 86.7% of cases, making insufficient access to specialist gynecological care an unlikely sole explanation for the delay.

Patient–physician communication before diagnosis was rated poorly overall, with a mean composite score of 2.96 on a 1–5 scale. The lowest-rated positive item was being informed about the possibility of endometriosis (mean 2.53), and the most frequently endorsed negative experience was being told that menstrual pain was “normal” (mean 3.49). The seven-item scale showed good internal consistency (Cronbach’s α = 0.836; 0.886 for the four positive items) and was suitable for factor analysis (KMO = 0.84; Bartlett’s test *p* < 0.001); it was unidimensional by Horn’s parallel analysis, which retained a single factor (first observed eigenvalue 3.79 vs. a 95th-percentile random value of 1.44; second 1.03 vs. 1.26), accounting for 54% of the variance (loadings 0.43–0.92). The two negatively worded items loaded most weakly (0.43–0.49), so the scale, although unidimensional, would benefit from further refinement. Item-level results are shown in Table 2.

Symptom minimization was reported by 76% of participants, unsatisfactory communication by 63%, and every respondent reported having been labeled at some point as anxious, hypersensitive, or exaggerating. Consistent with these ratings, 54% of women reported rarely or never feeling listened to (item 15), only 24.7% felt they had received clear explanations about their diagnosis and treatment (item 17), and 68% believed that better communication could have led to an earlier diagnosis (item 29).

**Figure 3:**
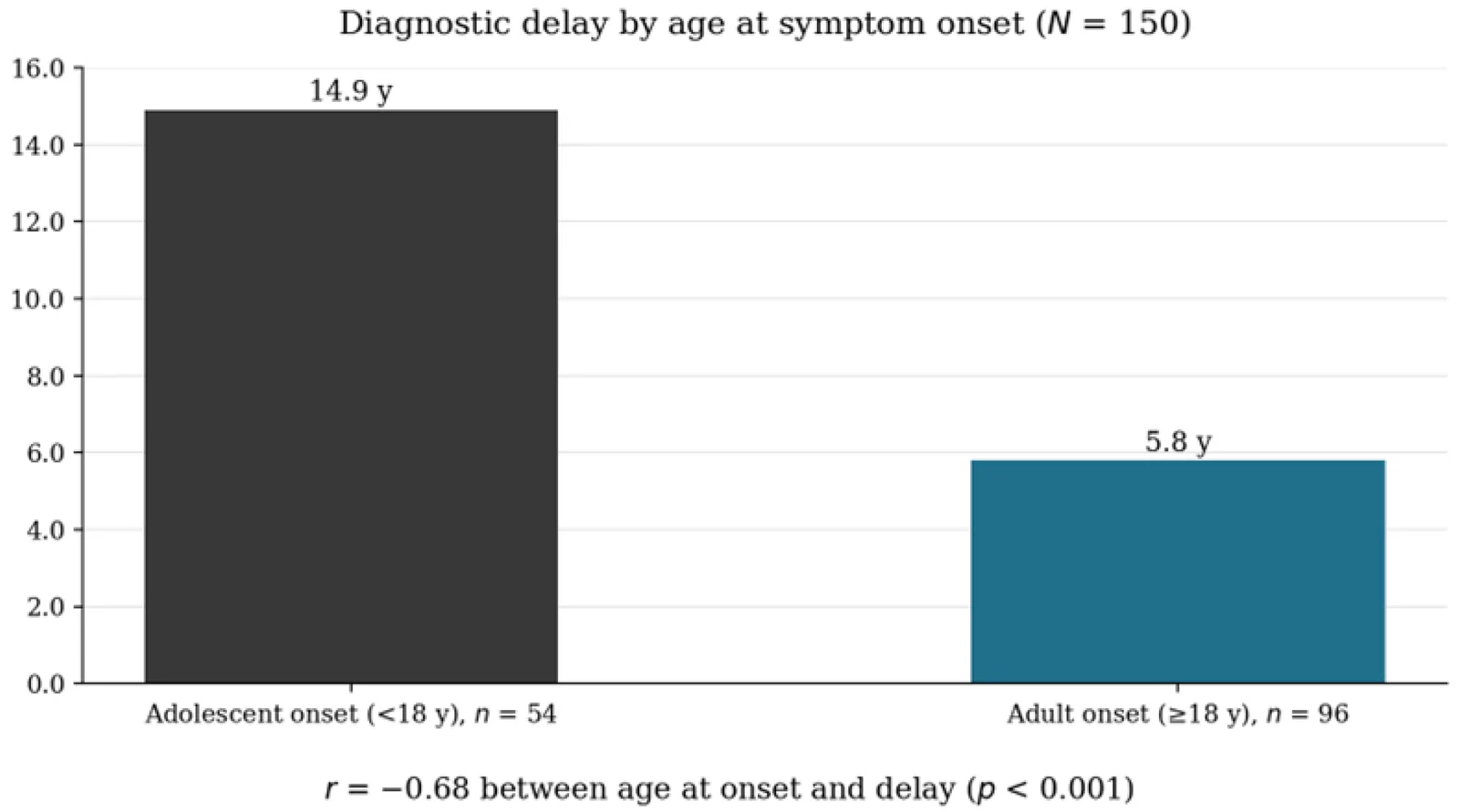
Mean diagnostic delay according to age at symptom onset (adolescent onset < 18 years vs. adult onset ≥ 18 years), *N* = 150.

Because diagnostic delay is derived arithmetically from the age at symptom onset, the association with age at onset is treated as partly structural and is examined separately in the dedicated sensitivity analyses reported below; the associations with the two care-pathway variables, patient–physician communication and the number of pre-diagnostic consultations, are the focus of this analysis. Diagnostic delay was most strongly related to the age at symptom onset (r = −0.68, 95% CI −0.76 to −0.58, *p* < 0.001): earlier onset was associated with a longer delay. Women whose symptoms began before 18 years of age waited a mean of 14.9 years for a diagnosis, compared with 5.8 years among those with adult-onset symptoms (Figure 3). Delay was also associated with a higher number of consultations (r = 0.33, 95% CI 0.18 to 0.47, *p* < 0.001), a lower communication score (r = 0.28, 95% CI −0.42 to −0.13, *p* < 0.001), and more deficient communication) (r = 0.26, 95% CI 0.10 to 0.40, *p* = 0.001). Symptom severity was not associated with delay (r = 0.07, 95% CI −0.09 to 0.23, *p* = 0.43) but was inversely related to the communication score (r = −0.28, 95% CI −0.42 to −0.13, *p* < 0.001). All of these associations remained significant after Benjamini–Hochberg correction for multiple comparisons (q < 0.05), whereas the null association between symptom severity and delay was unchanged (q = 0.43).

Women who reported that their symptoms had been minimized (*n* = 114) differed markedly from those who did not (*n* = 36): they had consulted more physicians (5.9 vs. 2.9, *p* < 0.001), attended more consultations (11.5 vs. 5.9, *p* < 0.001), reported higher symptom severity (8.3 vs. 6.7, *p* = 0.004), lower communication scores (2.54 vs. 4.30, *p* < 0.001; d = −2.23), and a longer diagnostic delay (10.0 vs. 6.0 years, *p* < 0.001; d = 0.60).

In a multivariable linear regression predicting diagnostic delay (R^2^ = 0.51, adjusted R^2^ = 0.49; F(5,144) = 29.5, *p* < 0.001), earlier age at symptom onset (b = −0.55, 95% CI −0.65 to −0.44, *p* < 0.001) and a higher number of consultations (b = 0.13, 95% CI 0.02 to 0.23, *p* = 0.021) were the only variables independently associated with delay; the communication score, symptom severity, and place of residence were not significant after adjustment (Table 3). The model explained about half of the variance in delay (adjusted R^2^ = 0.49) and met regression assumptions: all variance inflation factors were below 1.3, indicating no multicollinearity, and residuals were homoscedastic (Breusch-Pagan *p* = 0.06); residuals departed only mildly from normality (Shapiro–Wilk W = 0.97), which is unproblematic for linear regression at this sample size.

**Figure 4:**
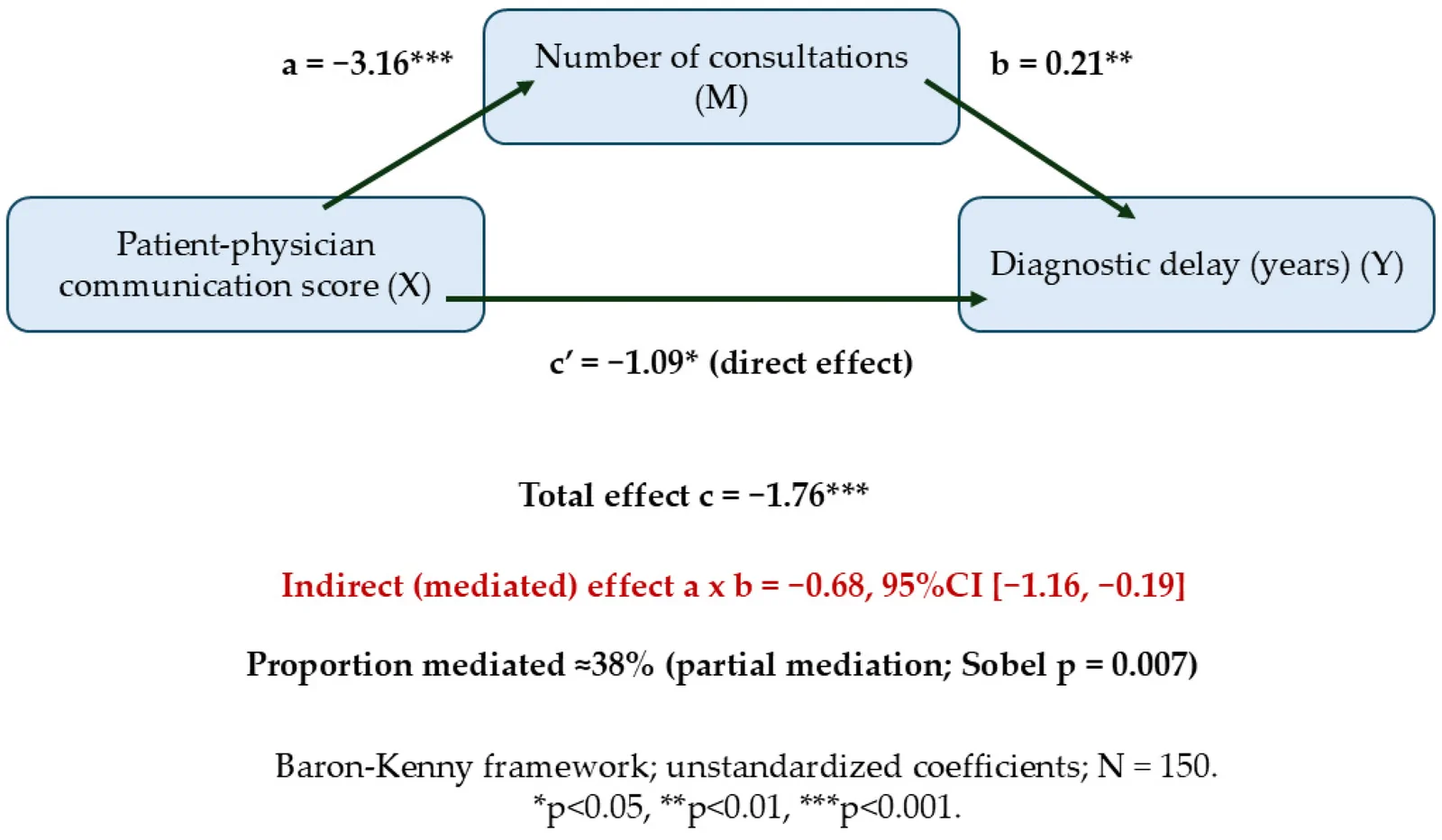
Mediation of the association between patient–physician communication and diagnostic delay by the number of consultations before diagnosis (Baron-Kenny framework; unstandardized coefficients; *N* = 150). The indirect effect was estimated by bias-corrected bootstrap (5000 resamples; 95% CI −1.26 to −0.22, primary estimate), with the Sobel test reported as a secondary estimate.

Because the communication score correlated with delay but did not remain significant after adjustment, we tested whether the number of consultations mediated this relationship (Figure 4). A higher communication score predicted fewer consultations (a = −3.16, *p* < 0.001), and more consultations predicted a longer delay after controlling for communication (b = 0.21, *p* = 0.002). The total effect of communication on delay (c = −1.76, *p* < 0.001) was reduced but remained significant when the mediator was included (c′ = −1.09, *p* = 0.042), indicating partial mediation. The indirect effect was significant (a × b = −0.68; bias-corrected bootstrap 95% CI −1.26 to −0.22, 5000 resamples; Sobel *p* = 0.007) and accounted for approximately 38% of the total effect. Because the design is cross-sectional, the reverse ordering, in which a longer diagnostic pathway itself generates more consultations, cannot be excluded; the mediator-outcome association persisted after adjustment for communication (partial r = 0.25, *p* = 0.002), but its direction cannot be established from these data. Coefficients for all mediation paths are reported in Table 4. The indirect effect was specific to the number of consultations: in a parallel-mediator model it operated through the number of consultations (−0.79; 95% CI −1.45 to −0.29) but not through the number of physicians (0.36; 95% CI −0.09 to 0.78). The same pathway was obtained for an alternative exposure, symptom minimization (indirect effect via consultations 1.30; 95% CI 0.45 to 2.38), and the effect was robust to winsorizing the consultation count at the 95th percentile (−0.65; 95% CI −1.23 to −0.20) and to excluding the single extreme value (−0.59).

Sensitivity analyses. Because diagnostic delay is derived arithmetically from the age at symptom onset, we repeated the analyses to verify that the care-pathway findings were not an artifact of this relationship. Women with adolescent-onset symptoms (<18 years) had a substantially longer delay than those with adult-onset symptoms (14.9 vs. 5.8 years; mean difference 9.1 years, 95% CI 7.2 to 11.0; Welch t = 9.50, *p* < 0.001; d = 1.67). When age at onset was removed from the multivariable model, a higher number of consultations (b = 0.21, *p* = 0.002) and a lower communication score (b = −1.14, *p* = 0.037) remained significant predictors of delay. Consistently, partial correlations controlling for age at onset confirmed that both the number of consultations (r = 0.24, *p* = 0.003) and the communication score (r = −0.17, *p* = 0.035) remained associated with delay. These analyses indicate that the associations of repeated healthcare encounters and communication with diagnostic delay are not explained by the structural link between age at onset and delay.

Exploratory comparisons by educational level and place of residence yielded only associations that were most plausibly attributable to the composition of the sample, which was predominantly urban and highly educated; to preserve focus, these analyses are reported in Supplementary Table S1.

Open-ended responses echoed the quantitative pattern. Many women described a gradual erosion of self-trust after repeated dismissal (“I started to believe that I was exaggerating and that I was probably overly sensitive to pain”), the normalization of severe pain (“I had come to normalize a nightmare that I was going through month after month since adolescence”), and disengagement from care (“there was a period when I refused to go to the doctor because it felt pointless”). For several, diagnosis brought relief and validation (“the day I finally received a diagnosis was also the day I had my last panic attack”).

When asked what had been most lacking in their interactions with physicians, participants most often cited professional competence (34%), followed by empathy (19%) and attentive listening (11%); a similar pattern emerged when women described what they would have wanted done differently, where professional competence (23%) and active listening (18%) again ranked highest. Alongside interpersonal aspects, therefore, perceived clinical competence in recognizing endometriosis emerged as a central unmet need.

## 4. Discussion

In this cross-sectional study of 150 Romanian women with self-reported, surgically confirmed endometriosis, the interval between symptom onset and diagnosis averaged 9.1 years (median 8; IQR 4–13). This figure is consistent with, and at the upper end of, the four-to-eleven-year range reported internationally [7,15] and with earlier European cohorts [8,16,17]. It confirms that, despite the shift toward non-surgical clinical diagnosis promoted by current guidelines [3], the diagnostic pathway for endometriosis remains long in our setting.

The strongest correlate of diagnostic delay was the age at which symptoms first appeared. Women whose symptoms began before 18 years waited on average 14.9 years for a diagnosis, compared with 5.8 years among those with adult-onset symptoms, and age at onset explained close to half of the variance in delay (r = −0.68). Because the delay interval is computed as the difference between age at diagnosis and age at onset, part of this association is arithmetic, and we therefore interpret early onset as a risk marker rather than a causal factor; sensitivity analyses excluding age at onset, together with partial correlations controlling for it, confirmed that the number of consultations and the communication score remained independently associated with delay. Even so, the direction and magnitude align with independent reports that younger age at symptom onset is associated with a longer delay [15], and with the well-documented under-recognition of endometriosis in adolescents, in whom dysmenorrhea is frequently dismissed as ordinary menstrual pain [7,18]. Adolescents with severe or persistent dysmenorrhea therefore represent a group in which a lower threshold for considering endometriosis could yield the largest reduction in delay.

Our central finding concerns how communication relates to delay. In the multivariable model, the communication score was not an independent predictor once age at onset and the number of consultations were taken into account, which cautions against treating poor communication as a direct cause of delay. The mediation analysis clarified the relationship: better communication was associated with fewer consultations (a = −3.16), more consultations were associated with a longer delay (b = 0.21), and the indirect path was significant (a × b = −0.68; bias-corrected bootstrap 95% CI −1.26 to −0.22 [primary estimate]; Sobel 95% CI −1.16 to −0.19; Sobel *p* = 0.007), accounting for roughly 38% of the total effect, although temporal ordering and causality cannot be inferred. Communication was associated with the diagnostic timeline largely through the number of consultations: women who felt less heard reported more consultations before the condition was recognized. Because the data are cross-sectional, the direction of this pathway cannot be established, and the reverse ordering, in which a longer diagnostic journey itself gathers more consultations, remains equally compatible with the data; we therefore treat the mediation as an associational, hypothesis-generating model rather than evidence of a causal mechanism. This interpretation is consistent with qualitative accounts in which repeated, unproductive consultations characterize the endometriosis diagnostic journey [8,16]. Consistent with this interpretation, 68% of the women themselves believed that better communication could have led to an earlier diagnosis, a patient-level perception that converges with the mediation result. The specificity of the association (through the number of consultations rather than the number of physicians), its reproduction with an alternative exposure, and its robustness to outliers make chance or a single influential observation an unlikely explanation, although none of these checks can resolve the direction of the association. Accordingly, we do not interpret the regression coefficient as a fixed per-consultation increment in delay: a longer diagnostic interval may itself generate additional consultations, and the questionnaire did not record the chronology, spacing, or purpose of individual encounters.

Symptom minimization emerged as a practical marker of this prolonged, repetitive diagnostic pathway. As reported in Section 3, the women who described their symptoms as minimized had a longer and more consultation-intensive diagnostic pathway and rated communication more poorly. That every respondent reported having been labeled at some point as anxious, hypersensitive, or exaggerating indicates that symptom invalidation was pervasive in this population, consistent with previous reports of gender bias in the assessment of women’s pain [19,20].

Symptom severity, by contrast, was not associated with diagnostic delay in either the correlation or the multivariable analysis. This tempers the intuitive expectation that more severe disease is recognized sooner: in our data, severity instead tracked with worse communication experiences (r = −0.28) and with symptom minimization. The dissociation between how much pain a woman reports and how quickly she is diagnosed is compatible with the understanding that endometriosis-associated pain is driven by sensitization mechanisms rather than by lesion burden [6], and it shifts attention from the intensity of symptoms toward how they are communicated and interpreted. Because severity was captured by a single retrospective rating of the impact of symptoms on daily life, the absence of an association should not be read as proof that symptom intensity is irrelevant to diagnostic recognition; it may also reflect the limits of retrospective, single-item measurement.

The communication difficulties described by our participants extend beyond isolated interpersonal failures. Women with endometriosis often struggle to translate a diffuse, embodied pain into terms that clinicians act upon [11,12], and feelings of being dismissed or disbelieved recur across settings and health systems [5,21,22,23]. The good internal consistency (α = 0.836) and essentially unidimensional structure (a single factor explaining 54% of the variance; KMO = 0.84) of our seven-item communication scale suggest that these experiences can be captured in a compact, quantifiable form, although two negatively worded items loaded weakly and a second factor was marginal, so the instrument was developed for this study and would require refinement and external validation before use as a clinical or quality indicator [24]; good internal consistency does not establish construct validity, and the scale may partly reflect a general negative evaluation of a prolonged diagnostic journey rather than communication alone.

These findings point to two complementary levers. First, structured, patient-centered tools such as pre-consultation symptom questionnaires and patient-reported outcome measures may help standardize symptom reporting and reduce the number of unproductive encounters that lengthen the pathway [25,26]. Second, clinician education aimed at recognizing endometriosis-suggestive symptoms, particularly in adolescents, and at avoiding the premature normalization of menstrual pain addresses the interpersonal roots of minimization [18]. Strengthening health literacy and information access may further support earlier help-seeking and more informed clinical encounters [27]. Importantly, when asked what had been most lacking, participants cited professional competence more often than empathy alone, which identifies clinical recognition skills, and not only interpersonal warmth, as a candidate target of such education.

To our knowledge, this is the first study to model the number of consultations as a mediator of the association between patient–physician communication and diagnostic delay in endometriosis; its contribution lies in this pathway framing rather than in the individual associations, several of which are already well established. Earlier national work addressed the psychosocial burden of the disease without focusing on the diagnostic encounter [28]. The reliance of many patients on online communities for validation and information, evident in our recruitment setting, reflects unmet needs that persist after clinical contact [29] and reinforces the psychological impact of prolonged diagnostic uncertainty [30,31].

Several limitations qualify these findings. The cross-sectional, retrospective design relies on self-reported recall of events that, for many women, occurred years earlier, which may introduce recall bias, and communication was assessed after diagnosis, so recollections may have been colored by the eventual outcome. Recruitment through social media and patient organizations produced a sample that was predominantly urban and highly educated, limiting generalizability; the counterintuitive associations of urban residence and higher education with longer delay were most plausibly artifacts of sample composition and are reported, with due caution, in Supplementary Table S1. Diagnosis was self-reported as surgically confirmed but could not be independently verified because of the anonymous design. Moreover, restricting eligibility to surgically confirmed disease may have selected women with more complex, symptomatic, or historically managed endometriosis, because current guidelines increasingly support clinical and imaging-based diagnosis without mandatory laparoscopy; this criterion may therefore have influenced the estimated delay and limits generalizability to women diagnosed without surgery. Finally, mediation tested on cross-sectional data cannot establish temporal or causal ordering; the proposed pathway should be regarded as a hypothesis to be confirmed in prospective studies that use validated instruments and encounter-level data. In addition, only about 30% of the women who opened the questionnaire (150 of 498) completed it, which may accentuate selection bias, and the reported frequencies of poor communication and symptom minimization may therefore be higher than in the broader population of women with endometriosis; residual confounding by unmeasured clinical and health-system factors also cannot be excluded.

Current symptom-based questionnaires and predictive models for endometriosis remain heterogeneous and are not yet fully validated as screening instruments [32,33]; such tools are therefore best framed as a testable strategy rather than an established means of shortening diagnostic delay. Similar diagnostic and communication gaps have been described for other female reproductive conditions; in polycystic ovary syndrome, for example, delayed diagnosis, consulting multiple health professionals, and inadequate information have been linked to dissatisfaction with care [34].

## 5. Conclusions

In this cross-sectional study of Romanian women with self-reported, surgically confirmed endometriosis, the interval from symptom onset to diagnosis averaged about nine years and was shaped less by the intensity of symptoms than by when symptoms began and by how the subsequent care was organized. Symptoms that started in adolescence identified the women at greatest risk of prolonged delay, and poor patient–physician communication was indirectly associated with the pathway, through a higher number of consultations before endometriosis was considered. Symptom minimization, a frequent and measurable feature of this prolonged, repetitive diagnostic pathway, and perceived clinical competence, more than empathy alone, were the unmet needs the women cited most often.

Because the study is cross-sectional and the sample was self-selected, these findings are hypothesis-generating rather than confirmatory: in this sample, poorer reported communication and a greater number of consultations were associated with a more prolonged diagnostic pathway, but the direction and causal nature of these relationships could not be established. They nonetheless point to two concrete, testable priorities for shortening the diagnostic pathway: a lower threshold for suspecting endometriosis in adolescents with severe or persistent dysmenorrhea, and the use of structured, patient-centered tools, such as pre-consultation symptom questionnaires, that may reduce repetitive, unproductive consultations. Prospective studies using validated instruments are needed to confirm the proposed pathway and to test whether these measures shorten the time to diagnosis.

## Figures and Tables

**Table 1:** Sociodemographic characteristics and diagnostic pathway of the participants (*N* = 150).

Characteristic	Value
Age, years—M (SD)	35.4 (6.2)
Age range, years	20–57
Education—n (%)	
Primary	4 (2.7)
Secondary	19 (12.7)
Higher	75 (50.0)
Postgraduate	52 (34.7)
Residence—n (%)	
Urban	124 (82.7)
Rural	26 (17.3)
Age at symptom onset, years—M (SD)	21.7 (7.9)
Age at diagnosis, years—M (SD)	30.8 (5.9)
Diagnostic delay, years—M (SD); median [IQR]	9.1 (6.9); 8 [4–13]
Delay > 5 years—n (%)	98 (65.3)
No. of physicians consulted—M (SD); median	5.2 (4.7); 4
No. of consultations—M (SD); median	10.2 (8.5); 10
Diagnosis established by a gynecologist—n (%)	130 (86.7)

**Table 2:** Patient–physician communication items and internal consistency of the scale (*N* = 150).

Item (1–5 Likert)	M	SD	% Endorsing (4–5)
The physician listened carefully (Q8)	3.00	1.37	35.3
My symptoms were taken seriously (Q9)	2.81	1.44	34.0
I received clear explanations (Q10)	2.79	1.50	35.3
I was told the pain was “normal” (Q11) ^a^	3.49	1.59	57.3
I was referred without clear explanation (Q12) ^a^	2.57	1.66	35.3
I was informed about possible endometriosis (Q13)	2.53	1.74	36.7
I trusted the physician (Q14)	3.67	1.38	62.0
Global communication score (7 items)	2.96	1.09	-

Note. ^a^ Negatively worded item, reverse-scored in the global score; for these items “% endorsing” reflects the proportion confirming the negative experience. Cronbach’s α = 0.836 (7 items); 0.886 (4 positive items).

**Table 3:** Multivariable linear regression predicting diagnostic delay (years).

Predictor	b	SE	t	*p*
Age at symptom onset	−0.545	0.054	−10.12	<0.001
No. of consultations	0.125	0.054	2.33	0.021
Communication score	−0.486	0.419	−1.16	0.249
Symptom severity	−0.053	0.182	−0.29	0.771
Urban vs. rural residence	−0.798	1.078	−0.74	0.461
Intercept	21.62	2.43	8.90	<0.001

Note. R^2^ = 0.506; adjusted R^2^ = 0.489; F (5,144) = 29.5, *p* < 0.001. Reference category for residence: urban. 95% CIs for the coefficients: age at symptom onset −0.65 to −0.44; number of consultations 0.02 to 0.23; communication score −1.31 to 0.34; symptom severity −0.41 to 0.31; rural vs. urban residence −2.93 to 1.33.

**Table 4:** Mediation analysis: number of consultations as a mediator between communication and diagnostic delay.

Path	Coefficient	SE	t/z	*p*
a: communication → no. consultations	−3.157	0.586	−5.39	<0.001
b: no. consultations → delay (adj. for X)	0.214	0.068	3.14	0.002
c: communication → delay (total effect)	−1.762	0.500	−3.52	<0.001
c′: communication → delay (direct effect)	−1.088	0.531	−2.05	0.042
a × b: indirect (mediated) effect	−0.675	0.249	−2.71	0.007

Note. Baron-Kenny framework; unstandardized coefficients; N = 150. Indirect effect: bias-corrected bootstrap 95% CI −1.26 to −0.22 (5000 resamples; primary estimate); Sobel 95% CI −1.16 to −0.19; proportion mediated ≈ 38% (partial mediation).

## Data Availability

The de-identified data and analysis code supporting the findings are available from the corresponding author upon reasonable request.

## Supplementary Materials

The following supporting information can be downloaded at: https://www.mdpi.com/article/10.3390/healthcare14193237/s1, Supplementary File S1 (STROBE Statement—Checklist for cross-sectional studies); Supplementary Table S1 (Exploratory comparisons of diagnostic delay and communication score by educational level and place of residence (N = 150)); Supplementary File S2 (Study questionnaire (English translation)).

## Abbreviations

The following abbreviations are used in this manuscript:

EFA	exploratory factor analysis
ESHRE	European Society of Human Reproduction and Embryology
FDR	false discovery rate
HSD	honestly significant difference (Tukey’s test)
IDEA	International Deep Endometriosis Analysis
KMO	Kaiser–Meyer–Olkin (measure of sampling adequacy)
STROBE	Strengthening the Reporting of Observational Studies in Epidemiology
