# Supplementary material for: Physician Communication, Repeated Healthcare Encounters, and Diagnostic Delay in Endometriosis: An Exploratory Cross-Sectional Analysis
Source: Healthcare (Basel). 2026 Oct 1;14(19):3237. doi: 10.3390/healthcare14193237 (PMC13665199; doi:10.3390/healthcare14193237)
Supplement: Supplementary file 1 [file healthcare-14-03237-s001.zip › Supplementary File S1.pdf]

Supplementary File S1

STROBE Statement — Checklist for cross-sectional studies

"Physician Communication, Repeated Healthcare Encounters, and Diagnostic Delay in Endometriosis: An Exploratory Cross-Sectional Analysis"

Section / Item	STROBE recommendation	Reported in (section)	Page
**Title & abstract — 1a**	Indicate the study design in the title or abstract	Title; Abstract, Methods (observational, cross-sectional)	1-2
**Title & abstract — 1b**	Balanced summary of what was done and found	Structured Abstract (Background/Methods/Results/Conclusions)	2
**Introduction**			
**Background/rationale — 2**	Scientific background and rationale	Introduction	2-3
**Objectives — 3**	State specific objectives / hypotheses	Introduction, final paragraph (three aims and the mediation hypothesis)	3
**Methods**			
**Study design — 4**	Present key elements of study design early	Section 2.1 (Study Design and Reporting)	3
**Setting — 5**	Setting, locations, relevant dates (recruitment, data collection)	Section 2.2 (recruitment period and online setting)	3-4
**Participants — 6**	Eligibility criteria, sources, methods of selection	Section 2.2 (eligibility, recruitment, exclusions); flow in Figure 1 and 2.2	4
**Variables — 7**	Define outcomes, exposures, predictors, confounders	Section 2.4 (Variables and Derived Measures)	5
**Data sources/measurement — 8**	Sources of data and measurement methods	Sections 2.3 and 2.4; scale reliability/validity in 2.5 and Table 2	4-6
**Bias — 9**	Describe efforts to address potential bias	Section 4 (Limitations): recall, selection, retrospective assessment; unverifiable diagnosis	11-12
**Study size — 10**	Explain how the study size was arrived at	Section 2.2 (convenience sample, all eligible respondents); precision statement in 2.5	5
**Quantitative variables — 11**	How quantitative variables were handled; groupings	Section 2.4 (delay continuous; onset <18/ ≥18; delay >5 y; communication reverse-coding)	5-6

**Statistical methods — 12a**	Methods including confounding control	Section 2.5 (Pearson; Welch t; multivariable linear regression)	6, 8
**Statistical methods — 12b**	Subgroups and interactions	Section 2.5 and Results; Supplementary Table S1	4
**Statistical methods — 12c**	How missing data were addressed	Section 2.2 (incomplete responses excluded; complete-case analysis)	4
**Statistical methods — 12d**	Sampling strategy accounting	Not applicable (non-probability online convenience sample); Section 2.2 and Limitations	4
**Statistical methods — 12e**	Sensitivity analyses	Section 2.5 and Results (model without age at onset; partial correlations; parallel-mediator and outlier robustness)	6, 8
**Results**			
**Participants — 13**	Numbers at each stage; non-participation; flow diagram	Section 2.2 and Results; Figure S (498→150)	4, 6
**Descriptive data — 14**	Characteristics; missing data per variable	Results (sample paragraph); Table 1	6-7
**Outcome data — 15**	Report numbers / summary measures of the outcome	Results (diagnostic delay descriptives); Table 1	6-7
**Main results — 16**	Unadjusted and adjusted estimates with CIs	Results (correlations; Table 3 regression; Table 4 mediation with bootstrap 95% CI)	7-8
**Other analyses — 17**	Subgroups, sensitivity analyses	Results (Sensitivity analyses; Figures 1–2); Supplementary Table S1	8
**Discussion**			
**Key results — 18**	Summarise key results with reference to objectives	Discussion, paragraphs 1–3	9-10
**Limitations — 19**	Limitations, sources of bias, direction/magnitude	Discussion, Limitations paragraph	11-12
**Interpretation — 20**	Cautious overall interpretation	Discussion (mediation as associational/hypothesis) and Conclusions	9-12
**Generalizability — 21**	External validity	Discussion, Limitations (online, urban, highly educated, self-selected sample)	11-12
**Other information**			
**Funding — 22**	Source of funding and role of funders	Back matter (Funding statement)	12

**An Explanation and Elaboration article discusses each checklist item (available at www.strobe-statement.org).*
