# Supplementary material for: Physician Communication, Repeated Healthcare Encounters, and Diagnostic Delay in Endometriosis: An Exploratory Cross-Sectional Analysis
Source: Healthcare (Basel). 2026 Oct 1;14(19):3237. doi: 10.3390/healthcare14193237 (PMC13665199; doi:10.3390/healthcare14193237)
Supplement: Supplementary file 1 [file healthcare-14-03237-s001.zip › Supplementary File S2.pdf]

Supplementary File S2. Study questionnaire (English translation)

"Physician Communication, Repeated Healthcare Encounters, and Diagnostic Delay in Endometriosis: An Exploratory Cross-Sectional Analysis"

Note. The questionnaire was administered online in Romanian; this is an English translation provided for review. It comprised 30 closed- and open-ended items. Items 8–14 were rated on a five-point Likert scale (1 = strongly disagree to 5 = strongly agree); items 11 and 12 are negatively worded and were reverse-coded when computing the composite communication score.

Introductory statement and informed consent

Dear participant, you are invited to take part in a scientific study evaluating how patient–physician communication influences diagnostic delay in endometriosis, conducted as part of a doctoral research project at the "Grigore T. Popa" University of Medicine and Pharmacy, Iași. Participation is voluntary, anonymous, and confidential; data will be used for scientific purposes only, and you may withdraw at any time without any consequence for your relationship with medical staff. Completion takes approximately 10–15 minutes. By continuing, you consent to participate in the study.

Section I. General (demographic) information

1. Age: ____ years.

2. Educational level:

- Primary
- Secondary
- Higher
- Postgraduate

3. Area of residence:

- Urban
- Rural

4. Total number of physicians consulted before diagnosis: ____

5. Medical specialties consulted before diagnosis:

- Gynecology
- Family medicine
- Internal medicine
- Psychiatry
- Other: ____

Section II. Symptoms and impact

6. What were the main symptoms that led you to consult a physician?

- Severe menstrual pain
- Chronic pelvic pain
- Digestive/urinary disturbances
- Other: ____

7. How severe was the impact of the symptoms on your daily life?

0 = not at all to 10 = extreme.

Section III. Experience with physicians before diagnosis

Items 8–14 were rated on a five-point Likert scale (1 = strongly disagree to 5 = strongly agree).

8. The physician listened to me carefully.

9. I felt that my symptoms were taken seriously.

10. The physician clearly explained which investigations or next steps would follow.

11. I was told that it is "normal" to have menstrual pain. (negatively worded, reverse-coded)

12. I was referred to other specialties without clear explanations. (negatively worded, reverse-coded)

13. I was informed about the possibility of having endometriosis.

14. I trusted the physician who examined me.

Section IV. Perception of patient–physician communication

15. Did you feel that the physician listened to you carefully?

- Never
- Rarely
- Sometimes
- Most of the time
- Always

16. Do you feel that your symptoms were minimized or ignored?

- Yes
- No

17. Did you receive clear explanations about the diagnosis and treatment?

- Yes

- Partially
- No

18. How would you characterize your communication with the physicians?

- Unsatisfactory
- Satisfactory
- Good
- Very good

19. What would you have wanted to happen differently in the communication? (open-ended)

Section V. Personal perception

20. What did you feel was most lacking in your communication with physicians before the diagnosis? (open-ended)

21. Have you ever been labeled as “hypersensitive”, “anxious”, or as “imagining the pain”?

- Yes
- No

22. If your answer to the previous question is YES, please specify whether and how you were affected. (open-ended)

23. What would you have wanted to happen differently in your relationship with the physicians before the diagnosis was established? (open-ended)

Section VI. Diagnosis and final outcome

24. Who was the physician who finally diagnosed endometriosis?

- Gynecologist
- Surgeon
- Family physician
- Internist
- Gastroenterologist
- Someone else

25. At what age did the symptoms suggestive of endometriosis begin? ____ years.

26. At what age was endometriosis diagnosed? ____ years.

27. Approximately how many medical consultations did you have before receiving the correct diagnosis? ____

28. How long passed from symptom onset to diagnosis?

- Less than 1 year
- 1–3 years
- 3–5 years
- More than 5 years

29. Do you consider that better communication would have led to a faster diagnosis?

- Yes
- No
- Do not know

Section VII. Other comments

30. Do you have any other observations regarding your experience up to diagnosis or the treatment received? (open-ended)
