# Supplementary material for: Physician Communication, Repeated Healthcare Encounters, and Diagnostic Delay in Endometriosis: An Exploratory Cross-Sectional Analysis
Source: Healthcare (Basel). 2026 Oct 1;14(19):3237. doi: 10.3390/healthcare14193237 (PMC13665199; doi:10.3390/healthcare14193237)
Supplement: Supplementary file 1 [file healthcare-14-03237-s001.zip › Supplementary Table S1.pdf]

Supplementary Table S1

"Physician Communication, Repeated Healthcare Encounters, and Diagnostic Delay in Endometriosis: An Exploratory Cross-Sectional Analysis"

Exploratory comparisons of diagnostic delay and communication score by educational level and place of residence (N = 150).

Subgroup	n	Diagnostic delay, years - M (SD)	Communication score - M (SD)
Educational level			
Primary	4	10.75 (2.06)	1.46 (0.21)
Secondary	19	5.16 (5.62)	3.49 (1.07)
Higher	75	10.16 (7.00)	2.87 (1.15)
Postgraduate	52	8.77 (6.96)	3.01 (0.92)
Place of residence			
Urban	124	9.56 (6.98)	2.94 (1.08)
Rural	26	6.69 (6.01)	3.07 (1.16)

Note. Values are means (SD). For diagnostic delay, one-way ANOVA across educational levels was significant ($F(3,146) = 2.88$, $p = 0.038$); only the secondary vs higher-education contrast was consistent across Tukey HSD and Bonferroni-corrected Welch comparisons ($d = -0.74$). For communication score, ANOVA was significant ($F(3,146) = 4.52$, $p = 0.005$), driven by lower scores in the primary-education group ($n = 4$). Urban vs rural delay differed on Welch's t-test ($p = 0.038$, $d = 0.42$). Given the small primary-education ($n = 4$) and rural ($n = 26$) subgroups and the predominantly urban, highly educated sample, these associations are exploratory and most plausibly reflect sample composition.
